# Environmental Radon Exposure and Inflammatory Responses in Children and Adolescents: Evidence from a High-Radon Region in Kazakhstan

**DOI:** 10.3390/biomedicines14051045

**Published:** 2026-05-04

**Authors:** Anel Lesbek, Yasutaka Omori, Meirat Bakhtin, Tomisato Miura, Shinji Tokonami, Polat Kazymbet, Danara Ibrayeva, Nursulu Altaeva, Baglan Kazhiyakhmetova, Elena Saifulina, Aigerim Shokabayeva, Elvira Mussayeva, Yelshenbek Mulkat, Yerlan Kashkinbayev

**Affiliations:** 1 Institute of Radiobiology and Radiation Protection NJSC, Astana Medical University, Astana 010000, Kazakhstan; bakhtin.m@amu.kz (M.B.); kazimbet.p@amu.kz (P.K.); danaraibrayeva@gmail.com (D.I.); kazhiyakhmetova.r@amu.kz (B.K.); saifulina.e@amu.kz (E.S.); sh_aika_88@mail.ru (A.S.); mussayeva.e@amu.kz (E.M.); yelshenbek.m@amu.kz (Y.M.); 2 Institute of Radiation Emergency Medicine, Hirosaki University, 66-1 Hon-cho, Hirosaki 036-8564, Japan; ys-omori@hirosaki-u.ac.jp (Y.O.); tomisato@hirosaki-u.ac.jp (T.M.); tokonami@hirosaki-u.ac.jp (S.T.); 3 Department of Medical Genetics and Molecular Biology NJSC, Astana Medical University, Astana 010000, Kazakhstan; altaeva.n@amu.kz

**Keywords:** radon exposure, environmental radiation, adolescents, inflammatory biomarkers, interleukin-8, Radon Exposure Index, environmental health, rural populations, serum biomarkers, Kazakhstan

## Abstract

**Background/Objectives:** Radon is a naturally occurring radioactive gas and the leading source of natural radiation exposure worldwide; however, its systemic biological effects in children remain poorly understood. This study examined the association between cumulative indoor radon exposure and inflammatory biomarkers among children residing in rural communities of the Aqmola region in Kazakhstan. **Methods:** The study included 87 children and adolescents (42 exposed and 45 controls). Radon exposure was measured in residential and school environments, and a composite Radon Exposure Index (REI) was constructed to estimate cumulative exposure over time. Serum concentrations of inflammatory biomarkers, including C-reactive protein (CRP), tumor necrosis factor-α (TNF-α), interleukin-1β (IL-1β), interleukin-6 (IL-6), and interleukin-8 (IL-8), were measured using validated immunoassay methods. Multivariable linear regression models adjusted for age, sex, body mass index, pubertal development stage, and heating type were used to evaluate associations between REI and biomarker levels. **Results:** Children and adolescents living in the radon-exposed community had significantly higher REI values than controls (7.75 ± 0.85 vs.4.83 ± 0.41, respectively). Among the biomarkers examined, CRP, TNF-α, IL-1β, IL-8 and IL-6 were not significantly associated with radon exposure. **Conclusions:** These findings do not support the use of the evaluated inflammatory biomarkers as indicators of early biological effects of environmental radon exposure in this population. However, the clear exposure contrast observed between study settings underscores the ongoing public health relevance of radon as an environmental hazard. Continued efforts to monitor and mitigate radon exposure in high-risk regions remain essential, particularly in environments where children spend substantial amounts of time.

## 1. Introduction

Environmental exposures during childhood and adolescence are critical determinants of health, as these periods involve ongoing physiological maturation and increased susceptibility to external stressors [1,2]. Among these exposures, ionizing radiation represents a biologically plausible trigger of early cellular and systemic responses, even at relatively low doses. Radon, a naturally occurring radioactive gas produced by the decay of uranium in soil and rock [3], is the primary source of background radiation exposure for the general population and is well established as the second leading cause of lung cancer after tobacco smoking [4,5,6].

Radon enters indoor environments such as homes and schools through soil–building interfaces and can accumulate to elevated concentrations depending on geological conditions, building characteristics, and ventilation patterns [7,8,9]. Children spend most of their time indoors, particularly in residential and school environments, cumulative exposure may occur across multiple microenvironments. Evidence from observational studies indicates that school-aged children (6–17 years) spend on average approximately 100 min per day outdoors, with outdoor time decreasing further during colder weather conditions [10]. This is particularly relevant in radon-prone regions, where prolonged exposure may begin early in life and persist throughout critical developmental stages.

In Kazakhstan, several regions are characterized by elevated environmental radon levels due to uranium-rich geological formations and historical mining activities. In particular, parts of the Aqmola region contain mineral-rich deposits and mining operations that disturb uranium-bearing rock strata, facilitating the release of radon into the surrounding environment [11,12,13,14]. In such settings, radon can accumulate not only in residential dwellings but also in public buildings such as schools and educational facilities [15]. Persistent elevated indoor radon concentrations have been documented in some rural settlements, where building characteristics and limited ventilation may further contribute to indoor radon accumulation [16,17]. Consequently, children in these areas may experience prolonged radon exposure across multiple indoor environments.

The health effects of radon exposure have been studied extensively in relation to lung cancer risk in adults [4,5,6,18,19]; however, much less is known about the broader biological effects of chronic exposure at concentrations above the World Health Organization (WHO) reference level of 100 Bq/m^3^, particularly in younger populations [20]. Ionizing radiation emitted by radon decay products can induce cellular damage through alpha particle emissions that interact with respiratory epithelium and surrounding tissues. Beyond carcinogenic mechanisms, radiation exposure has also been associated with inflammatory and immune responses that may contribute to systemic biological effects [21,22]. Emerging evidence suggests that inflammatory pathways may represent an important intermediary mechanism linking environmental radiation exposure with adverse health outcomes. Interestingly, radon exposure has also been explored in therapeutic contexts: controlled low-dose radon exposure is used in radon balneotherapy in several countries for the treatment of chronic inflammatory and musculoskeletal conditions [23]. However, studies examining inflammatory biomarkers in relation to chronic radon exposure in children remain limited and inconsistent.

Despite growing evidence linking radon exposure to adverse respiratory outcomes, there remains a limited understanding of its potential systemic biological effects, particularly in pediatric populations. Existing studies have primarily focused on lung cancer risk in adults [24,25,26,27], with relatively few investigations examining early biological responses to environmental radon exposure in children and adolescents. Moreover, prior research has rarely incorporated integrated exposure metrics that account for multiple indoor environments where children spend substantial time.

To address these gaps, the present study evaluates the association between cumulative indoor radon exposure—captured using a composite Radon Exposure Index (REI) integrating both residential and school environments—and circulating inflammatory biomarkers in children and adolescents residing in a high-radon region of Kazakhstan. The selection of inflammatory biomarkers in the present study (CRP, IL-1β, IL-6, IL-8 and TNF-α) was guided by their established roles in radiation-induced inflammatory and oxidative stress pathways. These biomarkers were selected to capture both early and downstream components of inflammatory signaling potentially triggered by chronic low-dose radiation exposure. By focusing on a vulnerable developmental population and applying a time-weighted exposure framework, this study aims to provide novel insights into potential early biological responses to chronic environmental radon exposure. These findings may contribute to improving risk assessment approaches and informing public health strategies in radon-prone settings.

## 2. Materials and Methods

### 2.1. Study Participants

This study included 87 children, comprising 42 cases and 45 controls. Children in the case group were recruited from Aqsu, a rural settlement in the Aqmola region characterized by elevated residential radon concentrations [13]. This settlement is located near the Aqsu mining area, a mineral-rich zone with active extraction of gold, platinum, palladium, and molybdenum. The surrounding territory includes two active open-pit mining sites, which are considered important contributors to increased environmental radon levels due to the disturbance of uranium-bearing geological formations and enhanced release of radon gas from underlying rocks and soils. The control group consisted of 45 children from another Aqmol village within the Aqmola region that does not contain mining operations or industrial extraction activities. Environmental monitoring has not identified elevated radon levels in this area, making it a suitable comparison population with similar regional and socioeconomic characteristics but without known radon exposure sources.

Baseline characteristics of the participants are presented in Table 1. Statistical comparisons indicated no significant differences between cases and controls in demographic or developmental variables, including gender distribution, age, body mass index (BMI), and pubertal development stage (PDS). Specifically, the proportion of males and females was comparable between groups (*p* = 0.441), and median age values were similar (14.0 years in cases vs. 13.0 years in controls; *p* = 0.246). Likewise, BMI and PDS did not differ significantly between the two groups (*p* = 0.986 and *p* = 0.813, respectively), indicating that the case and control populations were broadly comparable with respect to anthropometric and developmental characteristics. The only statistically significant difference observed between the groups concerned the type of residential heating system used in the households. In the case group, 78.6% of households relied on solid-fuel or stove-based heating systems, whereas the majority of control households (91.1%) used central heating system. This difference was statistically significant (*p* < 0.001), suggesting potential variation in indoor environmental conditions between the two communities. Overall, the similarity in key demographic and developmental characteristics between groups supports the comparability of the study populations and strengthens the interpretation of radon exposure as a primary environmental difference between the case and control settings.

### 2.2. Radon Measurement

Indoor radon progeny concentrations were assessed using semiconductor-based active monitoring systems. The equivalent equilibrium volumetric activity (EEVA) of radon decay products was measured using two complementary instruments: the RAMON-02 radiometer (Solo LLP, Kazakhstan) and the Alpharad Plus system (DOZA SPZ, Russian Federation). The RAMON-02 device quantifies alpha-emitting aerosol-bound radon progeny collected on a filter through controlled air sampling, while the Alpharad Plus system provides spectrometric detection of alpha emissions from short-lived progeny isotopes (e.g., polonium-218 and polonium-214) using a silicon detector. Measurements were conducted in frequently occupied indoor spaces (living rooms and bedrooms) under standardized conditions. Windows were kept closed for at least 30 min prior to and during measurement, and devices were positioned at breathing height (approximately 1–1.5 m above floor level) and away from direct airflow sources. Sampling was performed during daytime hours (9:00 AM–6:00 PM), with three consecutive measurements obtained at each location. Geometric means of these repeated measurements were calculated to reduce short-term variability. Measurements in school environments were conducted following the same protocol across different floors of each building. Radon progeny concentrations were assessed in multiple classrooms, and mean values across sampling locations were used to represent school-level exposure. The measurement systems provided estimates of EEVA, equilibrium factors, and related parameters describing indoor radon progeny behavior. These measurements were used to characterize environmental radiation conditions in residential and school settings and to support the assessment of indoor exposure. Indoor radon concentrations used for construction of the Radon Exposure Index (REI) were derived from these measurements and served as the primary exposure variable in the statistical analyses.

Measurements were conducted in 24 homes in Aqsu and 36 homes in Aqmol, as well as in classrooms attended by study participants. The number of residential measurement locations was lower than the number of study participants because multiple participants (children and adolescents) resided within the same household. In Aqsu, 42 participants were represented by 24 household measurement locations, and in Aqmol, 45 participants were represented by 36 households, reflecting typical household compositions with 2–3 children per residence. Each participant was assigned the EEVA value measured in their respective household. Within dwellings, measurements were focused on children’s bedrooms and living rooms as the principal residential occupancy areas; in schools, measurements were obtained in classrooms on different floors and averaged to characterize school-based exposure. To improve comparability across locations, measurements were performed under standardized indoor conditions, with instruments placed at 1.0–1.5 m above the floor, away from walls and ventilation sources, and after keeping windows closed for at least 30 min before sampling.

During the autumn–winter period, EEVA was measured in October (autumn) and in December–January (winter). In each home and classroom, three measurements were taken between 9:00 and 18:00, and arithmetic means were calculated. This approach improved the stability of exposure estimates. Further details on instrumentation, calibration, sampling procedures, and quality control have been published previously by the research team [14]. Measurements were conducted during the autumn–winter period (October and December–January), when indoor radon concentrations are typically elevated due to reduced ventilation and increased heating. As such, these measurements may approximate higher-end exposure conditions rather than annual averages.

### 2.3. Questionnaires and Biometrics

Anthropometric measurements were obtained during a scheduled study visit. Each participant’s height (in centimeters) and weight (in kilograms) were measured using standard procedures, and these values were used to calculate BMI for each child. BMI was computed as weight in kilograms divided by height in meters squared and used as an indicator of general anthropometric status in the study population. Pubertal maturation was assessed using the PDS. The questionnaire was completed by a parent or guardian. The questionnaire was administered in a private setting using a paper survey to ensure confidentiality and minimize response bias. A trained member of the research team was present to provide clarification if needed. PDS responses were used to derive a pubertal development stage following the scoring procedures described by Shirtcliff et al. [28], resulting in a composite score corresponding to Tanner developmental stages [29].

In addition, parents or children were asked to report characteristics of the home environment relevant to indoor exposure conditions. Specifically, information regarding the primary heating system used in the household was collected through a brief questionnaire. This variable was included to account for potential differences in indoor environmental conditions between households, as heating type may influence indoor air exchange patterns and consequently affect indoor radon concentrations.

### 2.4. Computing the Radon Exposure Index

Radon health effects are driven by cumulative exposure rather than short-term concentrations. Therefore, we constructed a composite REI to approximate each participant’s chronic exposure to indoor radon. The index integrates radon concentrations measured in the child’s primary microenvironments (home and school) with the estimated duration of exposure in those settings. Radon concentrations used for REI construction were derived from active measurements of EEVA of radon progeny obtained using semiconductor-based monitoring systems, as described in Section 2.2. Radon concentrations were expressed in becquerels per cubic meter (Bq/m^3^). These active measurements were used as the primary exposure data for both residential and school environments, ensuring methodological consistency across exposure assessments.

To account for time spent in different environments, exposure duration was weighted according to the proportion of time children typically spend at home versus school. Based on daily activity patterns for school-aged children, a weighting coefficient of 0.75 was assigned to the home environment and 0.25 to the school environment. Exposure duration at home was approximated by the child’s age in years multiplied by the home exposure coefficient, whereas school exposure duration was approximated using the current school grade multiplied by the school exposure coefficient. These parameters were used to estimate cumulative radon exposure in each setting. The Radon Exposure Index (REI) was calculated as the natural logarithm of the weighted sum of radon exposure in the home and school environments, with a constant added to avoid undefined values for zero exposure:

$$REI=ln([RC_{home}\times ET_{home}\times Co_{home}]+[RC_{school}\times ET_{school}\times Co_{school}]+1)$$

where $RC_{home}$ and $RC_{school}$ represent radon concentrations in the home and school environments, respectively, $ET_{home}$ and $ET_{school}$ denote exposure durations, and $Co_{home}$ and $Co_{school}$ represent weighting coefficients (0.75 and 0.25, respectively). These coefficients were applied once within the model to reflect the relative time spent in each environment.

These weights were based on the assumption that school-aged children spend approximately 5–6 h per day in school, consistent with standard public education schedules in Kazakhstan. This distribution is further supported by time-activity studies indicating that children spend the majority of their time indoors, with a substantial proportion occurring in the home environment outside school hours, particularly during colder seasons [30,31]. The resulting index was log-transformed to reduce right skewness and improve distributional properties for regression modeling, as environmental exposure variables such as indoor radon typically exhibit positively skewed distributions. This index provides a simplified proxy for cumulative radon exposure during early life, integrating both environmental concentration and exposure duration across major indoor settings relevant for children. A similar composite approach integrating environmental concentrations with time-activity patterns has been applied in previous studies of radon exposure in children to approximate cumulative exposure across multiple microenvironments, supporting the use of such indices in pediatric environmental epidemiology [22]. The REI should be interpreted as a simplified proxy of cumulative exposure rather than a validated dosimetric measure. It is based on assumptions regarding time–activity patterns and may not fully capture individual variability in exposure.

### 2.5. Serum Sampling and Laboratory Assessment

Blood samples were collected to assess inflammatory biomarkers potentially associated with environmental radon exposure. To minimize short-term variability and reduce potential interference from recent dietary intake, participants were instructed to fast for at least eight hours prior to sample collection. Venous blood samples were obtained under standardized conditions by trained medical personnel. Following collection, samples were centrifuged to separate serum and subsequently stored and processed in accordance with the manufacturers’ protocols for each assay.

Serum concentrations of inflammatory markers were determined using validated immunoassay-based laboratory methods. C-reactive protein (CRP) was measured using the Tina-quant C-Reactive Protein IV assay (V4.0, 2023-09), which has a measurement range of 0.6–350 mg/L, an analytical sensitivity of 0.3 mg/L, and a functional sensitivity of 0.6 mg/L. The assay demonstrated high analytical precision, with intra-assay coefficients of variation ranging from 1.3% to 3.5% and inter-assay variability between 1.7% and 2.5%.

Pro-inflammatory cytokines were quantified using high-sensitivity enzyme-linked immunosorbent assay (ELISA) kits suitable for detecting low concentrations in serum. Interleukin-1β (IL-1β) was measured using the BMS224-2HS ELISA kit, which has a detection range of 0.16–10 pg/mL, an analytical sensitivity of 0.05 pg/mL, and a functional sensitivity of 0.16 pg/mL. Interleukin-6 (IL-6) was measured using the BMS213-2HS ELISA kit, with a measurement range of 0.08–5.00 pg/mL, an analytical sensitivity of 0.03 pg/mL, and a functional sensitivity of 0.08 pg/mL. Interleukin-8 (IL-8) was quantified using the KAC1301 ELISA, which has a range of 0.591–375 pg/mL, analytical sensitivity of 1.1 pg/mL, and an estimated functional sensitivity of approximately 40 pg/mL. Tumor necrosis factor-α (TNF-α) concentrations were determined using the BMS223-2HS ELISA kit, which has a detection range of 0.31–20 pg/mL, analytical sensitivity of 0.13 pg/mL, and functional sensitivity of 0.31 pg/mL. The intra- and inter-assay coefficients of variation for all cytokine assays indicated acceptable analytical reproducibility.

For biomarker concentrations falling below the functional sensitivity threshold, values were substituted with one-half of the lower limit of quantification (LLOQ/2), a commonly used approach in environmental and biomarker studies to address left-censored data. This method allows retention of all observations in statistical analyses while minimizing bias associated with complete case exclusion. However, given the potential for distortion when a large proportion of values fall below the quantification limit, the impact of this approach was further evaluated through descriptive analyses and sensitivity analyses, including reporting the proportion of values below the functional sensitivity threshold and conducting restricted analyses where feasible. Biomarker distributions were inspected after natural log transformation to account for right skewness typical of inflammatory markers. Observations exceeding three standard deviations above the group mean on the log-transformed scale were considered extreme values and were excluded from subsequent analyses. The analytical characteristics of all assays used in the study are summarized in Table 2.

### 2.6. Variables and Statistical Analysis

The primary objective of the study was to evaluate the association between cumulative radon exposure and inflammatory biomarkers in children. The primary exposure variable was the Radon Exposure Index (REI), a composite indicator reflecting cumulative residential and school radon exposure. The primary outcomes were serum concentrations of inflammatory biomarkers, including C-reactive protein (CRP), tumor necrosis factor-α (TNF-α), interleukin-1β (IL-1β), interleukin-6 (IL-6), and interleukin-8 (IL-8).

The selection of inflammatory biomarkers was based on prior evidence linking ionizing radiation exposure to activation of pro-inflammatory cytokine pathways, including IL-6, TNF-α, and IL-1β, as well as chemokine-mediated responses (IL-8) and systemic inflammatory markers (CRP). These markers collectively represent biologically plausible pathways through which chronic low-dose radiation may exert systemic effects.

Prior to statistical modeling, descriptive analyses were performed to compare baseline characteristics between the case and control groups. Continuous variables were summarized using means and standard deviations (SD) or medians with interquartile ranges (IQR) depending on distributional properties, while categorical variables were presented as counts and percentages. Group comparisons were conducted using t-tests for normally distributed variables, Wilcoxon rank-sum tests for non-parametric variables, and chi-square tests for categorical variables.

Inflammatory biomarker concentrations exhibited right-skewed distributions; therefore, values were natural log–transformed using a log1p transformation to improve normality and stabilize variance prior to regression modeling. Descriptive statistics before and after transformation were examined to confirm improved distributional properties.

The primary association between radon exposure and inflammatory biomarkers was evaluated using multivariable linear regression models. In these models, each log-transformed biomarker served as the dependent variable, and the Radon Exposure Index was included as the main independent variable. A predefined core set of covariates was included in all models to account for potential confounding factors known to influence inflammatory processes in children. These covariates included age, BMI, PDS, gender, and heating type.

All regression models were therefore adjusted for age, BMI, gender, PDS, and heating type. Results were reported as regression coefficients (β) with corresponding 95% confidence intervals and *p*-values. To account for multiple comparisons across the five biomarker-specific models, *p*-values were additionally adjusted using the Benjamini–Hochberg false discovery rate (FDR) procedure. Both unadjusted and FDR-adjusted *p*-values are reported. Model fit was evaluated using the coefficient of determination (R^2^), adjusted R^2^, and Akaike Information Criterion (AIC) to provide additional context for model performance and robustness. All statistical analyses were conducted using R statistical software (R Foundation for Statistical Computing, Vienna, Austria, version 4.5.1) within the RStudio environment (Software, PBC, Boston, MA, USA, version 2025.9.0.387) [32,33].

### 2.7. Ethical Considerations

The study was approved by the Local Bioethics Committee of Astana Medical University (Decision No. 16, Meeting No. 10, 7 November 2023). Data collection, including questionnaire-based information, was conducted through parent or legal guardian reports for all participants regardless of age. Written informed consent was obtained from parents or legal guardians prior to participation. In addition, age-appropriate assent was obtained from children aged 14 years and older in accordance with ethical standards for research involving minors.

## 3. Results

Table 3 presents the comparison of radiation exposure and circulating inflammatory biomarkers between the radon-exposed and control groups. As expected, the case group had a substantially higher REI than the control group (7.75 [0.85] vs. 4.83 [0.41]), and this difference remained highly significant (*p* < 0.001), confirming clear exposure contrast between the study groups. No statistically significant between-group differences were observed for the inflammatory biomarkers evaluated. Although IL-8 levels tended to be higher in the radon-exposed group compared with controls (0.44 [0.44, 1.68] vs. 0.42 [0.40, 0.44]), this difference did not reach statistical significance (*p* = 0.057). Likewise, no significant differences were observed for CRP, TNF-α, IL-1β, or IL-6. The descriptive statistics of inflammatory biomarkers before and after natural logarithmic transformation are presented in Supplementary Table S1.

A substantial proportion of biomarker measurements fell below the assays’ functional sensitivity thresholds (Table 4). Specifically, 78.2% of CRP values, 81.6% of TNF-α values, and 78.2% of IL-1β values were below their respective quantification limits, indicating considerable left-censoring for these markers. In contrast, all IL-6 measurements were above the functional sensitivity threshold (0.08 pg/mL).

**Figure 1:**
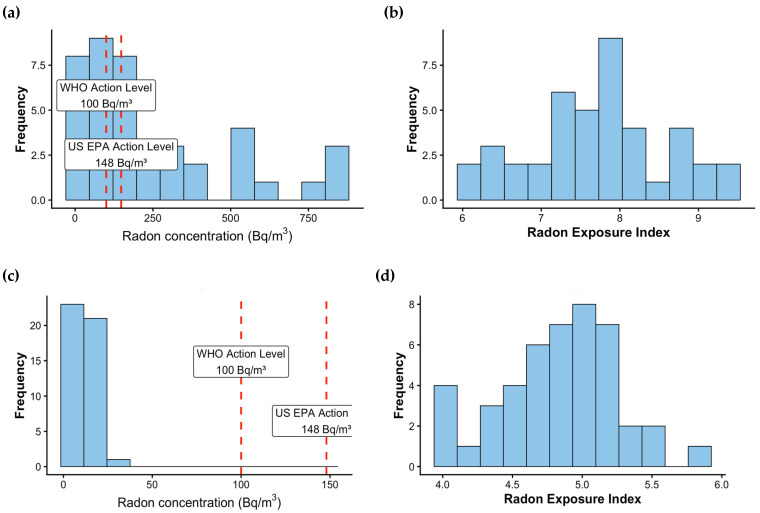
Distribution of Residential Radon Concentrations and Radon Exposure Index Among Children and Adolescents in Radon-Exposed and Control Communities. (**a**) Radon Concentration Level in Aqsu; (**b**) Radon Exposure Index in Aqsu; (**c**) Radon Concentration in Aqmol; (**d**) Radon Exposure Index in Aqmol.

Figure 1 illustrates the distribution of residential radon concentrations and REI among study participants from radon-exposed and control communities. Panel a presents histograms of residential radon concentrations measured in participants’ homes. In the radon-exposed community, radon concentrations demonstrated a wide distribution with several values exceeding international guideline thresholds. Dashed vertical lines indicate the World Health Organization (WHO) reference level of 100 Bq/m^3^ and the United States Environmental Protection Agency (US EPA) action level of 148 Bq/m^3^. A substantial proportion of measurements in the exposed community exceeded both thresholds, with some homes showing markedly elevated concentrations. In contrast, residential radon concentrations in the control community were generally lower and clustered below the WHO reference level, indicating substantially reduced environmental exposure. In addition, mean radon concentrations measured in schools were markedly elevated in the exposed area (417 Bq/m^3^) compared with the control community (9 Bq/m^3^), further indicating substantial differences in environmental radon exposure between the two settings.

Panel b displays the distribution of the REI, a composite indicator that integrates radon concentrations measured in the home and school environments with estimated exposure duration. Consistent with the residential measurements, REI values were higher and more broadly distributed in the radon-exposed community, reflecting greater cumulative exposure to indoor radon. In the control community, REI values were comparatively lower and showed a narrower distribution. Together, these distributions visually confirm the exposure contrast between the study populations and support the validity of the REI as a proxy measure for cumulative radon exposure in children and adolescents residing in radon-prone environments.

Table 5 presents the results of the adjusted linear regression analysis examining the association between the REI and circulating inflammatory biomarkers in the overall cohort (n = 87). Regression models were adjusted for potential confounders, including age, BMI, gender, PDS, and heating type. The analysis indicated that all inflammatory biomarkers were not significantly associated with REI. Specifically, no statistically significant associations were observed for CRP (β = 0.009, 95% CI: −0.053 to 0.072, *p* = 0.975), TNF-α (β = 0.007, 95% CI: −0.057 to 0.071, *p* = 0.975), IL-1β (β = 0.000, 95% CI: −0.022 to 0.023, *p* = 0.975), IL-6 (β = −0.147, 95% CI: −0.147 to 0.115, *p* = 0.975.) or IL-8 (β = 0.205, 95% CI: 0.009 to 0.400, *p* = 0.202). Sensitivity analyses using alternative REI weighting schemes are presented in Supplementary Table S2, and analyses addressing assay functional sensitivity limitations are provided in Supplementary Table S3.

**Figure 2:**
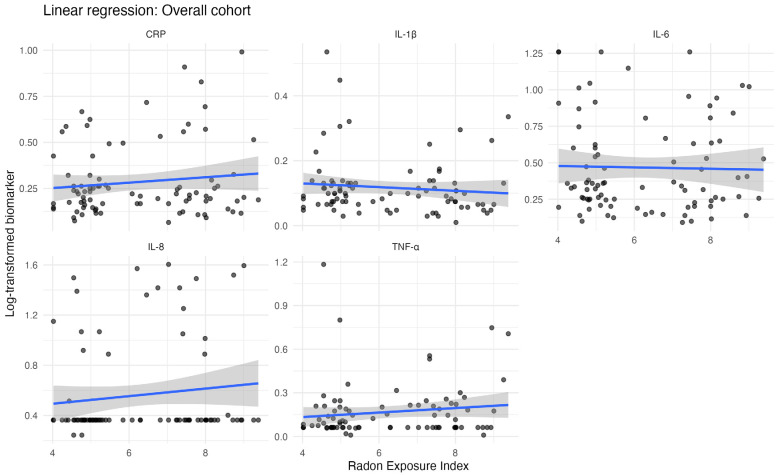
Overall regression plots of inflammatory biomarker with respect to Radon Exposure Index.

Figure 2 illustrates the corresponding regression plots for the association between the REI and each inflammatory biomarker in the overall cohort. Consistent with the regression results presented in Table 4, the plots indicate generally weak or flat associations between the REI and most biomarkers, including CRP, IL-1β, IL-6, TNF-α, and IL-8.

## 4. Discussion

This study examined the association between cumulative radon exposure and inflammatory biomarkers among children and adolescents living in rural communities with contrasting environmental radon levels. Several key findings emerged. First, adolescents residing in the radon-exposed settlement experienced substantially higher cumulative exposure to radon, reflected by markedly elevated REI values compared with controls. Second, all investigated biomarkers commonly associated with systemic inflammation—including CRP, TNF-α, IL-1β, IL-8 and IL-6—did not demonstrate statistically significant associations with the REI after adjustment for relevant covariates. Together, these findings suggest that chronic environmental radon exposure during adolescence is not associated with detectable or consistent alterations in circulating inflammatory biomarkers.

Inflammatory biomarkers are commonly used to assess systemic responses to environmental stressors, including radiation exposure. However, their interpretation in pediatric populations remains complex due to developmental variability and context-specific responses. Elevated levels of pro-inflammatory cytokines and chemokines have been linked to a range of adverse health outcomes, including respiratory diseases, metabolic dysregulation, cardiovascular risk factors, and neurodevelopmental alterations later in life [34,35]. Emerging evidence suggests that systemic inflammation during childhood and adolescence may be associated with reduced resilience to psychological stress and impaired cognitive functioning, potentially exerting long-term negative effects on neurodevelopment and mental health [36,37].

The absence of significant associations between radon exposure and inflammatory biomarkers is notable. Ionizing radiation emitted by radon decay products can generate reactive oxygen species and induce cellular damage in respiratory tissues, potentially activating inflammatory signaling pathways [38,39,40]. However, no consistent elevations in circulating inflammatory markers were observed in the present study. In this context, the absence of statistically significant and consistent associations between radon exposure and inflammatory biomarkers suggests that systemic inflammatory responses, if present, are likely to be small or not detectable using the current measurement approach. Although ionizing radiation may activate inflammatory pathways, our findings do not support this in the present cohort. Emerging evidence suggests that radon exposure may be associated with immune and inflammatory responses in children, including alterations in cytokine pathways relevant to airway inflammation and allergic disease [41,42]. Differences in study populations, exposure levels, biomarker selection, and measurement characteristics may contribute to the variability in findings across studies.

Our findings should be interpreted within the context of the broader literature examining radon exposure and inflammatory biomarkers. A recent systematic review and meta-analysis conducted by the same research group as the authors of the present study examined inflammatory biomarkers among individuals exposed to chronic residential or occupational radon [43]. The analysis reported that serum CRP and TNF-α levels were generally lower among adults with chronic radon exposure, suggesting a potential suppression of systemic inflammatory responses. The pooled analysis of multiple observational studies involving almost 35,000 participants indicated that mean CRP and TNF-α concentrations appeared reduced in radon-exposed adults. Several factors may explain this difference. First, age-related differences in immune function and inflammatory regulation may influence biological responses to environmental radiation. Second, radon-induced biological responses may vary depending on exposure duration and cumulative dose. It has been proposed that radon exposure may initially trigger inflammatory responses through oxidative stress and DNA damage, followed by longer-term immune modulation or suppression with prolonged exposure. Finally, differences in biomarker selection may also contribute to these discrepancies. These findings suggest that the relationship between radon exposure and inflammatory biomarkers is complex and context-dependent.

These findings have implications for public health interventions aimed at reducing radon exposure. However, given the absence of clear biological effects, these implications should be interpreted cautiously. Radon mitigation typically requires behavioral and environmental modifications at the household level, including improved ventilation, sealing of building foundations, and the installation of radon reduction systems. The Health Belief Model (HBM) provides a useful framework for understanding how individuals adopt such preventive actions, emphasizing the roles of perceived susceptibility, perceived severity, perceived benefits, and perceived barriers [44]. In the context of radon exposure, risk perception may be particularly limited, as radon is odorless, invisible, and does not produce immediate symptoms. Effective risk communication therefore remains central to radon prevention strategies by increasing awareness and promoting understanding of long-term health risks, particularly lung cancer, as well as the benefits of testing and mitigation [45,46].

Although the present study did not identify consistent or robust associations between radon exposure and circulating inflammatory biomarkers, it highlights the substantial exposure contrast in radon-prone settings and underscores the importance of environmental monitoring. Given the absence of clear biological signals and the methodological limitations related to biomarker measurement, these findings do not support the use of inflammatory biomarkers as indicators of early health effects in this context. Nevertheless, strengthening public awareness and promoting household-level radon testing and mitigation remain essential components of population-level risk reduction strategies.

This study has several limitations. First, the cross-sectional design precludes causal inference. Second, exposure assessment was based on short-term measurements conducted during the autumn–winter period, which may not reflect year-round variability in indoor radon concentrations. To improve exposure assessment, ongoing work includes long-term passive radon monitoring (commercial product: RADUET based on CR-39 detector, Radosys Ltd., Budapest, Hungary) [47]. Seasonal differences in ventilation and heating may therefore introduce exposure misclassification, and the Radon Exposure Index (REI) may represent elevated rather than average annual exposure levels. In addition, the REI is an approximate, non-validated proxy based on simplifying assumptions regarding time–activity patterns and exposure duration, which may further limit precision. Importantly, the REI represents a pragmatic approximation rather than a validated exposure metric, and its use may introduce exposure misclassification due to simplifying assumptions regarding time–activity patterns. Third, biomarker measurements were subject to analytical constraints. A substantial proportion of values, particularly for IL-8, fell below functional sensitivity thresholds, resulting in left-censoring and reliance on imputed values. Under these conditions, variation may reflect assay limitations rather than true biological differences. Although sensitivity analyses were conducted, they were limited by the small number of detectable observations. Fourth, the relatively small sample size may have reduced statistical power to detect modest associations, particularly after correction for multiple comparisons. Consequently, small or moderate effects cannot be excluded. Finally, differences in heating type between groups may reflect broader variation in housing characteristics, ventilation, and indoor exposures, and residual confounding from unmeasured environmental factors cannot be ruled out. Despite these limitations, the study provides a structured assessment of radon exposure across multiple indoor environments and contributes to the limited evidence on potential early biological responses to environmental radon exposure in children and adolescents.

This study offers several implications for environmental health policy and radon mitigation programs, although these should be interpreted with caution. First, the results underscore the importance of monitoring radon exposure not only in residential settings but also in schools and other environments where children spend substantial amounts of time, particularly in radon-prone regions. Second, the absence of consistent associations between radon exposure and circulating inflammatory biomarkers, together with the substantial proportion of values below assay sensitivity thresholds, suggests that the use of these biomarkers as indicators of early biological effects in this context may be limited. Third, although no robust evidence of systemic inflammatory alterations was observed, the clear exposure contrast between study populations reinforces the need for continued radon surveillance and mitigation efforts in high-risk areas. Public health strategies that integrate environmental monitoring, risk communication, and community-based interventions remain essential for reducing long-term exposure risks, particularly among vulnerable populations such as children.

## 5. Conclusions

In conclusion, this study demonstrated substantial differences in radon exposure between children and adolescents residing in radon-prone and control communities but found no consistent evidence of systemic inflammatory alterations associated with cumulative exposure. Across the panel of biomarkers examined, no statistically robust associations were observed after accounting for multiple comparisons, and the analysis was further limited by a high proportion of measurements below assay sensitivity thresholds for several markers.

These findings do not support the use of the evaluated inflammatory biomarkers as indicators of early biological effects of environmental radon exposure in this population. However, the clear exposure contrast observed between study settings underscores the ongoing public health relevance of radon as an environmental hazard. Continued monitoring and mitigation of radon exposure in high-risk regions remain essential.

Future research should prioritize longitudinal designs with improved exposure assessment, including long-term integrated radon measurements, and the use of more sensitive and biologically informative markers to better elucidate the potential health effects of chronic radon exposure during critical developmental periods.

## Figures and Tables

**Table 1:** Socio-demographic characteristics of the study participants.

Variable	Case	Control	*p*-Value
Gender	Male: 57.1% Female: 42.9%	Male: 48.9% Female: 51.1%	0.441
Age	14.00 [11.25, 15.00]	13.00 [11.00, 14.00]	0.246
BMI	18.89 [17.40, 19.98]	18.77 [17.36, 20.15]	0.986
PDS	4.00 [2.30, 4.00]	4.00 [2.60, 4.00]	0.813
Heating type	Central heating: 21.4% House heating: 78.6%	Central heating: 91.1% House heating: 8.9%	<0.001

Abbreviation: BMI—Body Mass Index; PDS—Pubertal Development Scale.

**Table 2:** Characteristics of Assays for Inflammatory Markers in the Radon Project.

Marker	Assay Type	Assay Range	Analytical Sensitivity	Functional Sensitivity	Intra Assay CV	Inter Assay CV
CRP	Tina-quant C-Reactive Protein IV (V4.0, 2023-09)	0.6–350 mg/L	0.3 mg/L	0.6 mg/L	1.3–3.5%	1.7–2.5%
IL-1β	ELISA (BMS224-2HS)	0.16–10 pg/mL	0.05 pg/mL	0.16 pg/mL	6.7%	3–9%
IL-6	ELISA (BMS213-2HS)	0.08–5.00 pg/mL	0.03 pg/mL	0.08 pg/mL	4.9%	3–8%
IL-8	ELISA (KAC1301)	0.591–375 pg/mL	1.1 pg/mL	~40 pg/mL	3.2–3.6%	8.6–13.1%
TNF-α	ELISA (BMS223-2HS)	0.31–20 pg/mL	0.13 pg/mL	0.31 pg/mL	8.5%	9.8%

Abbreviations: CV—coefficient of variation; CRP—C-reactive protein; ELISA—enzyme-linked immunosorbent assay; IL-1β—interleukin-1 beta; IL-6—interleukin-6; IL-8—interleukin-8; TNF-α—tumor necrosis factor alpha.

**Table 3:** Comparison of Inflammatory Biomarker Levels Between the Radon-Exposed and Control Groups.

Variable	Case	Control	*p*-Value
REI	7.75 (0.85)	4.83 (0.41)	<0.001
CRP	0.23 [0.18, 0.37]	0.26 [0.16, 0.35]	0.949
TNF-α	0.16 [0.06, 0.25]	0.09 [0.06, 0.19]	0.179
IL-1β	0.09 [0.05, 0.14]	0.11 [0.08, 0.14]	0.092
IL-6	0.40 [0.22, 0.91]	0.43 [0.29, 0.82]	0.572
IL-8	0.44 [0.44, 1.68]	0.42 [0.40, 0.44]	0.057

Data are presented as mean (SD) for REI and median [IQR] for inflammatory biomarkers; *p*-values were adjusted using the Benjamini–Hochberg false discovery rate procedure. Abbreviations: CRP—C-reactive protein; IL-1β—interleukin-1 beta; IL-6—interleukin-6; IL-8—interleukin-8; TNF-α—tumor necrosis factor alpha.

**Table 4:** Functional sensitivity and proportion of biomarker values below reliable assay range.

Biomarker	Functional Sensitivity	Below Functional Sensitivity, *n* (%)	Median [IQR]
CRP	0.60	68 (78.2%)	0.28 [0.20, 0.44]
TNF-α	0.31	71 (81.6%)	0.10 [0.07, 0.26]
IL-1β	0.16	68 (78.2%)	0.11 [0.07, 0.15]
IL-6	0.08	0 (0.0%)	0.51 [0.32, 1.42]
IL-8	40.00	84 (96.6%)	0.55 [0.55, 0.55]

**Table 5:** Adjusted Linear Regression between REI and Inflammatory Biomarkers.

Biomarker ^1^	n	Beta ^2^	95% CI	Unadjusted *p*-Value	FDR-Adjusted *p*-Value	R ^2^	Adjusted R ^2^	AIC
CRP	87	0.009	−0.053 to 0.072	0.767	0.975	0.107	0.040	53.2
TNF-α	87	0.007	−0.057 to 0.071	0.833	0.975	0.035	−0.037	57.7
IL-1β	87	0.000	−0.022 to 0.023	0.975	0.975	0.038	−0.034	−121.7
IL-6	87	0.016	−0.147 to 0.115	0.810	0.975	0.018	−0.056	182.2
IL-8	87	0.205	0.009 to 0.400	0.040	0.202	0.140	0.076	251.7

^1^ Biomarkers log-transformed using log1p. ^2^ Models adjusted for age, BMI, gender, PDS and heating type. Abbreviations: CRP—C-reactive protein; IL-1β—interleukin-1 beta; IL-6—interleukin-6; IL-8—interleukin-8; TNF-α—tumor necrosis factor alpha.

## Data Availability

The data presented in this study are available upon request from the first author, Anel Lesbek, and the correspondence author, Yerlan Kashkinbayev.

## Supplementary Materials

The following supporting information can be downloaded at: https://www.mdpi.com/article/10.3390/biomedicines14051045/s1, Table S1: Descriptive statistics for each of the inflammatory markers (pg/mL) before and after natural log transformation; Table S2. Sensitivity analysis using alternative REI weighting coefficients; Table S3. Sensitivity analyses addressing functional sensitivity concerns.

## Abbreviations

The following abbreviations are used in this manuscript:

BMI	Body Mass Index
Bq/m^3^	Becquerels per cubic meter
CRP	C-reactive protein
CV	Coefficient of variation
EEVA	Equivalent equilibrium volumetric activity
ELISA	Enzyme-linked immunosorbent assay
HBM	Health Belief Model
IL-1β	Interleukin-1 beta
IL-6	Interleukin-6
IL-8	Interleukin-8
IQR	Interquartile range
LLOQ	Lower limit of quantification
PDS	Pubertal Development Scale
REI	Radon Exposure Index
SD	Standard deviation
TNF-α	Tumor necrosis factor alpha
US EPA	United States Environmental Protection Agency
WHO	World Health Organization
