# Supplementary material for: Environmental Radon Exposure and Inflammatory Responses in Children and Adolescents: Evidence from a High-Radon Region in Kazakhstan
Source: Biomedicines. 2026 May 4;14(5):1045. doi: 10.3390/biomedicines14051045 (PMC13203900; doi:10.3390/biomedicines14051045)
Supplement: Supplementary file 1 [file biomedicines-14-01045-s001.zip › biomedicines-4283160-supplementary.pdf]

Table S1. Descriptive statistics for each of the inflammatory markers (pg/mL) before and after natural log transformation.

Marker	Orig Mean	Orig SD	Orig Range	Log Mean	Log SD	Log Range
CRP	0.53	0.70	0.07 – 4.44	0.36	0.32	0.07 – 1.69
IL1	0.15	0.15	0.01 – 1.03	0.13	0.11	0.01 – 0.71
IL6	1.60	2.61	0.1 – 11.47	0.69	0.64	0.1 – 2.52
IL8	4.71	10.92	0.32 – 52.21	0.93	1.02	0.28 – 3.97
TNF	0.34	0.98	0.01 – 8.64	0.21	0.31	0.01 – 2.27

Abbreviations: CV - coefficient of variation; CRP - C-reactive protein; ELISA - enzyme-linked immunosorbent assay; IL-1β - interleukin-1 beta; IL-6 - interleukin-6; IL-8 - interleukin-8; TNF-α - tumor necrosis factor alpha.

Table S2. Sensitivity analysis using alternative REI weighting coefficients.

REI weighting	Biomarker	n	Beta	95% CI	Unadjusted p-value	FDR-adjusted p-value
Home 0.75 / School 0.25	CRP	87	0.009	-0.053 to 0.072	0.767	0.975
Home 0.75 / School 0.25	TNF-α	87	0.007	-0.057 to 0.071	0.833	0.975
Home 0.75 / School 0.25	IL-1β	87	0.000	-0.022 to 0.023	0.975	0.975
Home 0.75 / School 0.25	IL-6	87	-0.016	-0.147 to 0.115	0.810	0.975
Home 0.75 / School 0.25	IL-8	87	0.205	0.009 to 0.400	0.040	0.202
Home 0.60 / School 0.40	CRP	87	0.008	-0.052 to 0.069	0.784	0.948
Home 0.60 / School 0.40	TNF-α	87	0.006	-0.056 to 0.068	0.845	0.948
Home 0.60 / School 0.40	IL-1β	87	-0.001	-0.023 to 0.021	0.948	0.948
Home 0.60 / School 0.40	IL-6	87	-0.026	-0.153 to 0.100	0.683	0.948
Home 0.60 / School 0.40	IL-8	87	0.216	0.029 to 0.404	0.025	0.123
Home 0.70 / School 0.20 / Outdoor 0.10	CRP	87	0.009	-0.054 to 0.072	0.769	0.945
Home 0.70 / School 0.20 / Outdoor 0.10	TNF-α	87	0.007	-0.058 to 0.072	0.830	0.945
Home 0.70 / School 0.20 / Outdoor 0.10	IL-1β	87	0.001	-0.022 to 0.024	0.945	0.945
Home 0.70 / School 0.20 / Outdoor 0.10	IL-6	87	-0.011	-0.144 to 0.121	0.864	0.945
Home 0.70 / School 0.20 / Outdoor 0.10	IL-8	87	0.198	0.000 to 0.396	0.050	0.248

Table S3. Sensitivity analyses addressing functional sensitivity concerns.

Biomarker	Analysis	Values above functional sensitivity, n (%)	Estimate	95% CI	p-value
CRP	Logistic regression: value ≥ functional sensitivity	19/87 (21.8%)	0.958	0.554 to 1.619	0.873
CRP	Linear regression restricted to values ≥ functional sensitivity	19/87 (21.8%)	Not estimated	Too few observations above functional sensitivity	Not estimated
TNF-α	Logistic regression: value ≥ functional sensitivity	16/87 (18.4%)	1.242	0.756 to 2.050	0.387

Biomarker	Analysis	Values above functional sensitivity, n (%)	Estimate	95% CI	p-value
TNF-α	Linear regression restricted to values ≥ functional sensitivity	16/87 (18.4%)	Not estimated	Too few observations above functional sensitivity	Not estimated
IL-1β	Logistic regression: value ≥ functional sensitivity	19/87 (21.8%)	0.890	0.533 to 1.438	0.639
IL-1β	Linear regression restricted to values ≥ functional sensitivity	19/87 (21.8%)	Not estimated	Too few observations above functional sensitivity	Not estimated
IL-6	Logistic regression: value ≥ functional sensitivity	87/87 (100.0%)	1.110	0.740 to 1.674	0.612
IL-6	Linear regression restricted to values ≥ functional sensitivity	87/87 (100.0%)	-0.016	-0.147 to 0.115	0.810
IL-8	Logistic regression: value ≥ functional sensitivity	3/87 (3.4%)	Not estimated	Insufficient values above/below threshold	Not estimated
IL-8	Linear regression restricted to values ≥ functional sensitivity	3/87 (3.4%)	Not estimated	Too few observations above functional sensitivity	Not estimated
